# Higher outdoor mosquito density and *Plasmodium* infection rates in and around malaria index case households in low transmission settings of Ethiopia: Implications for vector control

**DOI:** 10.1186/s13071-023-06088-2

**Published:** 2024-02-06

**Authors:** Ashenafi Abossie, Assalif Demissew, Hallelujah Getachew, Arega Tsegaye, Teshome Degefa, Kassahun Habtamu, Daibin Zhong, Xiaoming Wang, Ming-Chieh Lee, Guofa Zhou, Christopher L. King, James W. Kazura, Guiyun Yan, Delenasaw Yewhalaw

**Affiliations:** 1 Department of Medical Laboratory Sciences, College of Medicine and Health Sciences, Arba Minch University, Arba Minch, Ethiopia; 2 School of Medical Laboratory Sciences, Faculty of Health Sciences, Jimma University, Jimma, Ethiopia; 3 Department of Medical Laboratory Sciences, College of Medicine and Health Sciences, Ambo University, Ambo, Ethiopia; 4 Aklilu Lemma Institute of Pathobiology, Addis Ababa University, Addis Ababa, Ethiopia; 5 Department of Medical Laboratory Technology, Arba Minch College of Health Sciences, Arba Minch, Ethiopia; 6 Department of Biology, College of Natural Science, Jimma University, Jimma, Ethiopia; 7 Menelik II College of Health Sciences, Addis Ababa, Ethiopia; 8 Department of Microbial, Cellular and Molecular Biology, College of Natural Sciences, Addis Ababa University, Addis Ababa, Ethiopia; 9 Program in Public Health, University of California at Irvine, Irvine, CA 92697 USA; 10 Center for Global Health & Diseases, School of Medicine, Case Western Reserve University, Cleveland, 44106 OH USA; 11 Tropical and Infectious Diseases Research Center (TIDRC), Jimma University, Jimma, Ethiopia

**Keywords:** Index case, Reactive case detection, Sporozoite rate, Residual malaria, Ethiopia

## Abstract

**Background:**

Understanding the clustering of infections for persistent malaria transmission is critical to determining how and where to target specific interventions. This study aimed to determine the density, blood meal sources and malaria transmission risk of anopheline vectors by targeting malaria index cases, their neighboring households and control villages in Arjo-Didessa, southwestern Ethiopia.

**Methods:**

An entomological study was conducted concurrently with a reactive case detection (RCD) study from November 2019 to October 2021 in Arjo Didessa and the surrounding vicinity, southwestern Ethiopia. Anopheline mosquitoes were collected indoors and outdoors in index case households and their surrounding households (neighboring households), as well as in control households, using pyrethrum spray cache (PSC) and U.S. Centers for Disease Control and Prevention (CDC) light traps. Adult mosquitoes were morphologically identified, and speciation in the* Anopheles gambiae* complex was done by PCR. Mosquito *Plasmodium* infections and host blood meal sources were detected by circumsporozoite protein enzyme-linked immunosorbent assay (CSP-ELISA) and cytochrome *b*-based blood meal PCR, respectively.

**Results:**

Among the 770 anopheline mosquitoes collected, *An. gambiae* sensu lato (*A. gambiae* s.l.) was the predominant species, accounting for 87.1% (*n* = 671/770) of the catch, followed by the *Anopheles* *coustani* complex and *Anopheles pharoensis*, which accounted for 12.6% (*n* = 97/770) and 0.26% (*n* = 2/770) of the catch, respectively. From the sub-samples of *An. gambiae* s.l.analyzed with PCR, *An. arabiensis* and *Anopheles amharicus* were identified. The overall mean density of mosquitoes was 1.26 mosquitoes per trap per night using the CDC light traps. Outdoor mosquito density was significantly higher than indoor mosquito density in the index and neighboring households (*P* = 0.0001). The human blood index (HBI) and bovine blood index (BBI) of *An. arabiensis* were 20.8% (*n* = 34/168) and 24.0% (*n* = 41/168), respectively. The overall *Plasmodium* sporozoite infection rate of anophelines (*An. arabiensis and An. coustani* complex) was 4.4% (*n* = 34/770). Sporozoites were detected indoors and outdoors in captured anopheline mosquitoes. Of these CSP-positive species for *Pv-210*, *Pv-247* and *Pf*, 41.1% (*n* = 14/34) were captured outdoors. A significantly higher proportion of sporozoite-infected mosquitoes were caught in index case households (5.6%, *n* = 8/141) compared to control households (1.1%, *n* = 2/181) (*P* = 0.02), and in neighboring households (5.3%, *n* = 24/448) compared to control households (*P* = 0.01).

**Conclusions:**

The findings of this study indicated that malaria index cases and their neighboring households had higher outdoor mosquito densities and *Plasmodium* infection rates. The study also highlighted a relatively higher outdoor mosquito density, which could increase the potential risk of outdoor malaria transmission and may play a role in residual malaria transmission. Thus, it is important to strengthen the implementation of vector control interventions, such as targeted indoor residual spraying, long-lasting insecticidal nets and other supplementary vector control measures such as larval source management and community engagement approaches. Furthermore, in low transmission settings, such as the Arjo Didessa Sugarcane Plantation, providing health education to local communities, enhanced environmental management and entomological surveillance, along with case detection and management by targeting of malaria index cases and their immediate neighboring households, could be important measures to control residual malaria transmission and achieve the targeted elimination goals.

**Graphical Abstract:**

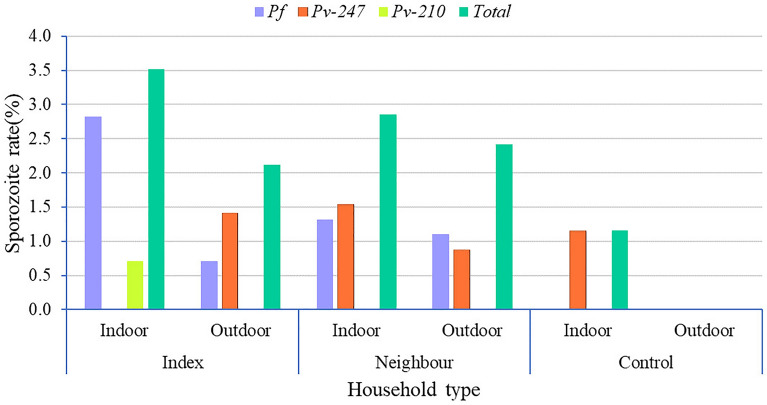

**Supplementary Information:**

The online version contains supplementary material available at 10.1186/s13071-023-06088-2.

## Background

In malaria control and elimination efforts, the use of interventions such as long-lasting insecticide-treated nets (LLINs), indoor residual spraying (IRS), artemisinin-based combination therapy (ACT) and rapid diagnostic tests (RDTs) has led to a significant reduction in the number of malaria cases and deaths [1]. Despite maintaining these core malaria vector control interventions, however, human and vector behaviorial changes are still significant challenges and contribute to ongoing malaria transmission, slowing the efforts  of malaria elimination in low transmission settings [2, 3].

In Ethiopia, the overall goal is to eliminate malaria nationwide by 2030. According to the national malaria program, between 2016 and 2019, there was a significant decline in malaria morbidity and mortality. During the same time frame, there a was 37% decline in annual parasite incidence (API) per 1000 population, from 19/1000 population to 12/1000. This has been achieved by expanding testing and treatment services across the country, by scaling up key malaria interventions, such as LLINs, through mass campaigns targeted at malaria-risk populations and by implementing  IRS in areas that are epidemic prone [4]. However, the progress faced disruption due to COVID-19 pandemic in 2020 [5].

Among the strategies of the National Malaria Program, interrupting residual malaria transmission is a target in low and very low transmission settings by achieving effective control of parasite reservoirs in the human host and vectors. This strategy includes the use of LLINs, targeted IRS and other supplementary vector control such as larval source management, as well as early case detection and management that can reduce the number of mosquitoes that transmit malaria and halt residual malaria transmission [6]. Despite good access to and usage of vector control interventions, residual malaria transmission could still occurs in low transmission settings [3]. Persistent residual malaria transmission can also occur due to various reasons, such as vector and human behaviors [7], the presence of asymptomatic carriers [8] and limitations of vector control impact, as well as failure of the implementation of vector control [9].

Since their initial implementation, the primary vector control interventions, such as LLINs and IRS, have significantly reduced malaria transmission [10]. However, the effectiveness of vector control interventions is being challenged by the emergence of insecticide resistance [11–13]. Current evidence has also shown that primary malaria vectors are resistant to pyrethroid [14, 15]. It has also been shown that the malaria vectors that have developed insecticide resistance are a result of selective pressures associated with agricultural pesticides [16].

In addition, indoor vector control may cause changes in the biting and resting behaviors of mosquitoes, alter species composition and increase the significance of secondary or local vectors [17, 18]. Regarding behavioral modification, the change in mosquito resting and feeding habits to outdoor resting and biting is the most challenging aspect of vector control [19, 20], which has an impact on the transmission of residual malaria [7]. Therefore, current indoor-based vector control efforts are ineffective against mosquitoes that bite and rest outdoors and can  substantially contribute to residual malaria transmission. However, Govella et al. [21] reported results suggesting that indoor insecticidal intervention could tackle both indoor and outdoor mosquitoes.

Malaria transmission can be localized, clustered or hotspot as well as heterogeneous over small areas in low transmission settings. In malaria control elimination efforts, the development of a tailored and targeted approach is needed to identify residual malaria transmission foci [22]. In this regard, reactive case detection (RCD) is a malaria surveillance approach that has been conducted by targeting the index case and neighboring households to detect residual malaria [23, 24]. Studies have investigated strategies that improve RCD in the detection of residual malaria transmission to achieve malaria elimination [25, 26]. Thus, entomological surveillance along with RCD is required to target specific populations and areas pockets of malaria transmission. This is crucial for vector control intervention in conjunction with the investigation of foci and management of cases to interrupt residual malaria transmission.

The Ministry of Health (MOH) of Ethiopia launched a subnational malaria elimination campaign in many districts in 2017 [6]. Arjo-Didessa Sugarcane Plantation and the surrounding districts are among the districts targeted for malaria elimination. This entomological survey was carried out in Arjo Didessa Sugarcane Plantation and the surrounding vicinity, in Southwestern Ethiopia. According to a retrospective study, between 2008 and 2017, there was a decrease in malaria positivity [27]. Furthermore, the prevalence of malaria parasites was 2.0% in 2019 [28]. LLIN coverage was proportional to the national and regional households in 2020 [29]. Therefore, this study aimed to determine the density, blood meal sources, and malaria transmission risk of anopheline vectors by targeting malaria index cases, their neighboring households, and control villages in Arjo-Didessa, Southwestern Ethiopia.

## Methods

### Study areas

**Fig. 1:**
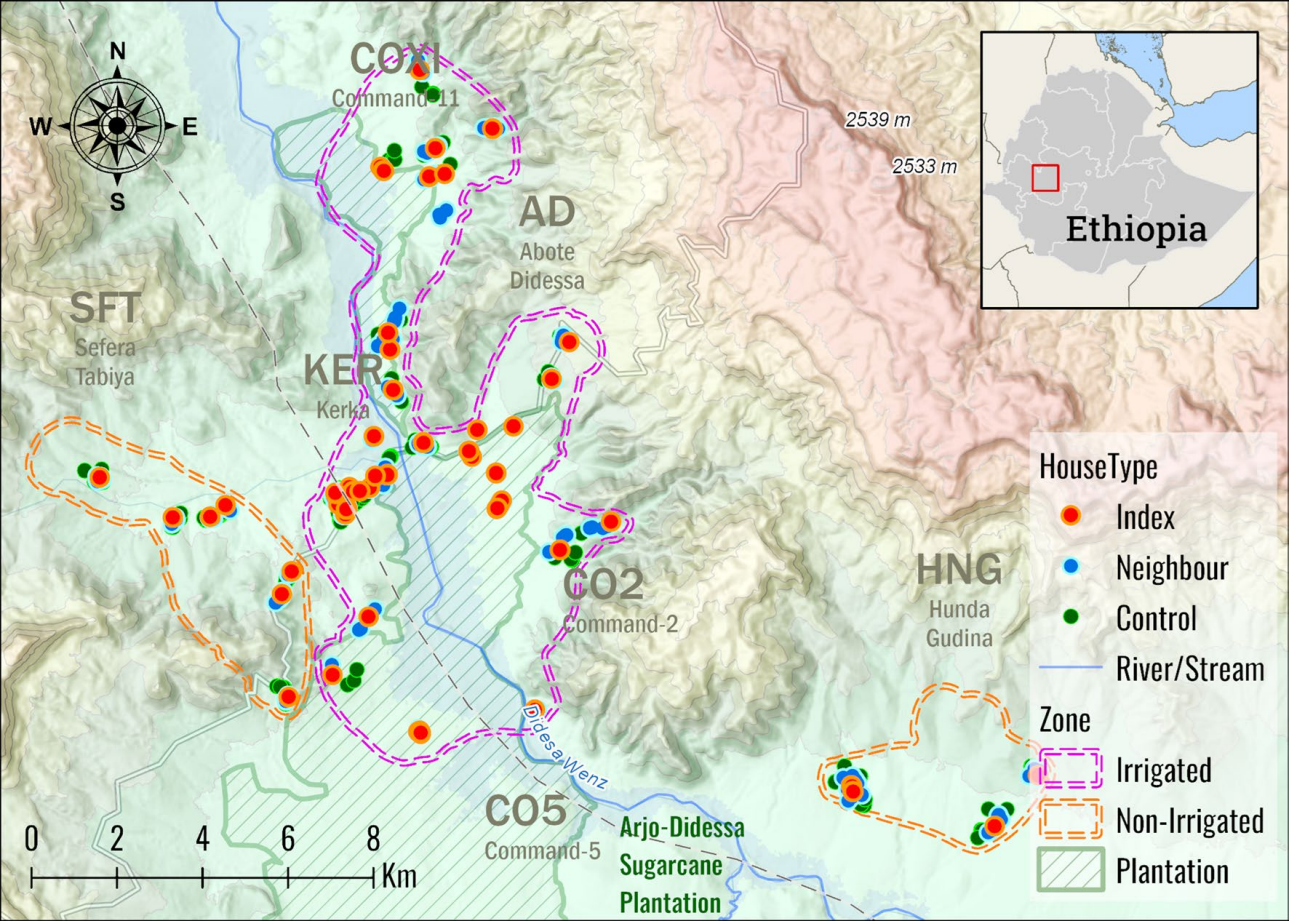
Map of the study area, Jimma Arjo and Dabo Hana districts, Ethiopia

The study was carried out in the catchment area of Arjo Didessa Sugarcane Plantation and the surrounding vicinity, in southwestern Ethiopia. Arjo-Didessa Sugar Cane Plantation area is located in Jimma Arjo district of East Wollega Zone and Dabo Hana district of Buno Bedele Zone (Fig. 1).

Arjo-Didessa Sugar Cane Plantation is located at 395 km distance along Addis Ababa-Nekemte route or 540 km distance on Addis Ababa-Jimma-Bedelle-Nekemte route. The study covered eight villages or clusters included in Ethiopia project study areas of the International Center of Excellence for Malaria Research (ICEMR). The study area (8°41′35.5″N and 36°25′54.9″E) is located at altitudes ranging from 1300 to 2280 m a.s.l. The districts have two malaria transmission seasons: peak malaria incidence occurs between September and December following the main rainy season of June to September, and low transmission occurs from April to May during and after a short rainy season in February to March. The remaining months of the year are dry months. In the present study, the eight villages or clusters targeted for the entomological study were divided into two different ecological zones: irrigated clusters (Command 2 [CO2], Command 5 [CO5], Command 11 [CO11], Abote Didessa [AD] and Kerka [KER] clusters) and non-irrigated clusters (Hundie Gudina [HG], Soyoma [HNG] and Sefera Tabiya [SFT] clusters).

Jimma Arjo and Dabo Hana districts are bordered by the Didessa River, which flows through the districts in the study area. Arjo-Didessa Sugarcane Plantation uses water from the river through small canals, drainage and water sprinklers for watering the seedlings. The majority of the population in the area are farmers who cultivate maize (*Zea mays*) and pepper (genus *Capsicum*) crops, and the remaing population are workers at sugarcane plantations and factories. The local population also raises animals, such as cattle, sheep, goats, horses, donkeys and chickens. Most of the houses in the village are made of mud or wood only, with grass-thatched roofs. Animal pens are adjacent to residents’ houses. Factory workers live in houses made of brick blocks or iron sheet walls with corrugated iron roofs.

*Anopheles arabiensis* is considered to be the major vector in the study area. Among members of the *An. gambiae* complex, *Anopheles amharicus* co-occurs in the study area [14]. *Plasmodium vivax* is the predominant malaria species [30], and a retrospective study showed that there has been a remarkable reduction in overall malaria infection in 10 years [27]. Another recent study also showed no significant difference in malaria prevalence in irrigated and non-irrigated clusters of the study area. However, the prevalence of malaria significantly differs by season and is higher in the wet season, both in irrigated and non-irrigated sites [28].

### Entomological study

The entomological study was conducted from November 2019 to October 2021 concurrently with the RCD study. The household selection was based on the *P. vivax* index cases who were willing to participate in both the RCD and entomological studies. *Plasmodium vivax* index cases were recruited from eight passive case detection (PCD) clinics in Arjo Didessa, and they were traced back to their household level. For a single *P. vivax* index case household, approximately five randomly selected neighboring and five control households, located at a radius of 1 to 200 m and 201 to 500 m distant from the *P. vivax* index case households, respectively, were included in the study according to previous studies elsewhere [14, 23].

The present study included a total of 52 *P. vivax* index case households, 216 neighboring households and 135 control households. For a single index case, including the index case household, U.S. Centers for Disease Control and Prevention light traps (CDC-LTs; John Hock Company, Gainesville, FL, USA) were installed in 11 households for 3 consecutive months for adult mosquito collection. CDC-LTs were set indoors and outdoors in the selected RCD households. Traps were set 1.5 m above the ground at the foot end of the bed with an LLIN, while the outdoor CDC-LTs were hung next to the houses approximately 50 cm above the ground. The traps were operated from 6:00 p.m. to 6:00 a.m.

Indoor resting mosquitoes were collected using pyrethrum spray catches (PSCs) following a standard protocol [31] using insecticide aerosol (BAYGON^®^; S. C. Johnson & Son, Racine, WI, USA). PSCs were conducted from 6:00 a.m. to 8:00 a.m., for 3 consecutive months. When collecting mosquitoes using PSCs, prior to spraying with the insecticide aerosol, the occupants were requested to leave the house; utensils used for food, drinking water and clothes were taken out of the houses; doors were covered with cloth to prevent mosquitoes from escaping during spraying; and the floor of the house was covered with white sheets. After closing the doors and windows, the insecticide aerosol was sprayed in the room. Ten minutes after spraying, the white sheet was removed from the house, and knocked-down mosquitoes were then collected and preserved individually in 1.5-ml Eppendorf tubes containing silica gel desiccant. The mapping of households was done by the Global Positioning System (GPS), and the geographical coordinates were recorded.

### Laboratory investigations

#### Morphological identification of mosquitoes

All female mosquitoes collected from households were transported to the Arjo ICEMR laboratory, killed by freezing and identified to species level morphologically, according to Gillies and Coetzee [32]. Blood-feeding status was visually assessed for each mosquito, and each mosquito was sorted as unfed, fed, half gravid or gravid. All female anopheline mosquitoes were individually preserved in labeled Eppendorf tubes containing silica gel and stored at − 20 ºC, until further processing.

#### Mosquito processing and DNA extraction

Anopheline mosquitoes were processed at the Tropical Infectious Diseases Research Centre (TIDRC) laboratory at Jimma University, Sokoru, Ethiopia. Body parts of each mosquito, such as head/thorax, abdomen and wings/legs, were separated for sporozoite detection, blood meal analysis and molecular mosquito identification to confirm species, respectively.

DNA was extracted using a modified Chelex-100 resin [33]. Briefly, a homogenized sample, 950 µl of phosphate-buffered saline (PBS) and 50 µl of 10% saponin (Sigma-Aldrich, St. Louis, MO, USA) were added to an Eppendorf tube and incubated at 4 ºC for > 4 h or overnight. Following incubation, the mixture was centrifuged at 14,000 rpm for 10 min at room temperature, and the supernatant was discarded. After removing any remnants, 1000 µl of PBS was added to the Eppendorf tube and the mixture centrifuged at 14,000 rpm for 5 min. Again, the supernatant was discarded and the remaining contents of the tube spun for 30 s. Finally, the remaining liquid was removed using a 200-µl pipet. After the sample in the tube was air dried for 15 min, 150 µl of 20% Chelex resin (Sigma-Aldrich) suspension and 100 µl of ddH_2_O were added to the dried sample and incubated at 95 ºC in a water bath for 10 min, with mixing by vortex every 2 min. The mixture was then centrifuged at 1400 rpm for 1 min, and the extracted DNA was transferred into Nunc tubes and preserved at − 20 °C until molecular analysis.

#### *Anopheles gambiae* complex identification by PCR

Sub-samples of the *An. gambiae* complex were selected for PCR analysis to determine the species as described by Scott et al. [34]. A total reaction volume of 25 μl of PCR mix containing 0.5 μl of each primer (Universal [UN], *An. gambiae* [GA], *An. arabiensis* [AR] and *An. amharicus* [QD], 2 μl of genomic DNA extracted from a single mosquito, 12.5 μl Green Taq PCR Master Mix (2×) (Thermo Fisher Scientific, Waltham, MA, USA) and nuclease-free water for amplification. The primer sequences were 5′-GTGTGCCCCTTCCTCGATGT-3′ (UN); 5′-CTGGTTTGGTCGGCACGTTT-3′ (GA); 5′-AAGTGTCCTTCTCCATCCTA-3′ (AR); and 5′–CAGACCAAGATGGTTAGTAT-3′ (QD). PCR cycling was performed with an initial step at 5 min at 95 °C to activate the DNA polymerase; followed by 30 cycles of denaturation for 30 s at 94 °C, annealing for 30 s at 50 °C and extension for 30 s at 72 °C; with a final extension for 10 min at 72 °C. The species-specific nucleotide sequences in the ribosomal DNA were used to identify the *An. gambiae* complex using three deferentially sized amplicons. *Anopheles gambiae* (390 bp), *An. arabiensis* (315 bp) and *An. amharicus* (150 bp) were the species-extracted product sizes. A UV trans-illuminator was used to visualize the amplified DNA in a 2.0% agarose gel stained with ethidium bromide.

#### Molecular identification of mosquito blood meal source by multiplex PCR

All engorged mosquitoes were chosen as specimens to detect host DNA using multiplex PCR. A molecular analysis was carried out on each mosquito. One universal reverse primer and five vertebrate host-specific forward primers (human, pig, bovine, goat and dog) were used to amplify the cytochrome* b* gene, which is encoded in the mitochondrial genome, to test for the specific host origin of the blood meal using conventional PCR. Primers and their sequences for the cytochrome * b*-based PCR blood meal identification assay were: UnRev1025 (5′–GGTTGTCCTCCAATTCATGTTA–3′), Pig573F (5′–CTCGCAGCCGTACATCTC–3′), Human741F (5′–GGCTTACTTCTTCATTCCTCCT–3′) Goat894F (5′–CCTAATCTTAGTACTTGTACCCTTCCTC–3′), Cow121F (5′–CATCGGCACAAATTTAGTCG–3′) and Dog368F (5′–GAATTGTACTATTATTCGAACCAT–3′) [35, 36].

Species-specific primers were used to detect each host DNA in the samples. PCR amplification for human, bovine, pig, goat and dog samples was carried out in a reaction volume of 25 µl containing 12.5 µl of Taq Master Mix 2× (Promega, Madison, WI, USA), 0.5 µl of each primer (10 µM), 1–2 µl of DNA and nuclease-free water. Positive DNA samples of humans, bovines, goats and dogs were used as positive controls for each specific primer, while double-distilled water was used as the negative control in each PCR run. The PCR cycling conditions for the amplification of DNA were one cycle at 95 °C for 5 min; followed by 40 cycles at 95 °C for 60 s, 56 °C for 60 s and 72 °C for 60 s; with a final cycle at 72 °C for 7 min. The PCR yields fragments that indicate the following species: human (334 bp), pig (453 bp)*,* goat (132 bp)*,* dog (680, bp) and cow (561 bp). Following amplification, the PCR products were separated and visualized by electrophoresis in a 2.0% agarose gel with ethidium bromide running buffer. The results were compared to a 100-bp DNA ladder by placing the agarose gel on a UV transilluminator (UVP, LLC, Upland, CA, USA).

#### Sporozoite infection

Anopheline mosquito heads and thoraces were analyzed by circumsporozoite protein enzyme-linked immunosorbent assay (CSP-ELISA) for the detection of *Plasmodium* sporozoite parasites, as explained by Ljungstrom et al. [36]. All female *An*. *gambiae* complex specimens were dissected, and the heads and thoraces were analyzed for the presence of  CSP of  *P. falciparum*, *P. vivax*-210 and *P. vivax*-247 CSP ELISA kit (Malaria Research and Reference Reagent Resource Center [MR4], ATCC, Manassas, VA, USA) at the TIDRC laboratory in Sekoru, Jimma University. For the detection of *Plasmodium* infection, the CSP-ELISA was performed on all *An. gambiae* complex samples individually, whereas a pool of five samples was run for the *Anopheles coustani* complex.

Briefly, the head and thorax of an *Anopheles* mosquitoes were ground in a labeled 1.5-ml Eppendorf tube. Then, 50 μl of the captured monoclonal antibodies (MAbs) of *P*. *falciparum* (*Pf*), *P. vivax* (*Pv*-210) and *P. vivax* (*Pv*-247) was added to labeled 96-well plates run in duplicate. The plates were covered with aluminum foil and incubated at room temperature for 30 min. After incubation, well contents were decanted and the plates struck 5 times onto a paper towel, following which 200 μl blocking buffer was added again to each well; the wells were then covered and the plates incubated for 1 h. The well contents were then aspirated and struck 5 times onto a paper towel, following which 50 μl of each mosquito triturate (including positive and negative control) was added; the wells were again covered with aluminum foil and the plates incubated for 2 h. The mosquito triturates were then aspirated from each well and washed twice with 200 μl PBS-Tween-20 using ELISA-Washer (ELx800; BioTek, Winooski, VT, USA). After this step, 50 μl of peroxidase-linked MAbs of *Pf*, *Pv-*210 and *Pv*-247 was added; the wells were covered with aluminum foil and the plates incubated for 1 h. The plates were then washed 3 times with 200 μl of PBS-Tween-20. A substrate solution of 100 μl ABTs A and B was added, and the plates were incubated for 30 min. Finally, the plates were read after 30 min and 1 h for *Pf* and *Pv,* respectively, at 405–411 nm absorbance using the ELISA plate reader. Specimens were considered positive if the absorbance of the individual well was twofold higher than the absorbance of the negative control samples at 405–411 nm.

### Data analysis

All data collected were recorded in electronic forms using a tablet in which an Open Data Kit (ODK) was installed and entered into Microsoft Excel (Microsoft Corp., Redmond, WA, USA). Data analysis was done using STATA software version 17.0 (Stata Corp. College Station, TX, USA), and JMP© Pro version 16.0.0 (SAS Institute Inc., Cary, NC, USA). Log transformation was done to normalize the data [log 10(*x* + 1)]. Variations in the mean among RCD household types were analyzed using a one-way analysis of variance (ANOVA) (*P* < 0.05). The post hoc test was performed using Tukey’s Kramer HSD method. Chi-square tests (*χ*^2^) and Fisher’s exact tests were used to determine whether there was a significant difference between more than two proportions and between two proportions, respectively. The t-test was used to compare the mean difference in mosquito density between indoor and outdoor locations. The density of* Anopheles* mosquitoes was calculated as the number of female *Anopheles* mosquitoes per trap per night for the CDC-LT collection method. The sporozoite rates were calculated as the number of mosquitoes testing positive for sporozoites divided by the total number of mosquitoes tested. The human blood index (HBI) and bovine blood index (BBI) were calculated by dividing the number of mosquitoes that obtained their meals from humans and bovines (including mixed blood meal origins) by the total number of blood-fed *Anopheles* mosquitoes analyzed. In all statistical tests, values were considered to be significantly different if *P* < 0.05.

## Results

### Anopheline mosquito composition and density

A total of 576 trap nights using CDC-LTs and 357 PSCs were set for mosquito collection, resulting in the capture of 770 anopheline mosquitoes. Of these 770 mosquitoes, 94.2% (*n* = 725/770) were captured by CDC-LTs and 5.8% (*n* = 45/770) by PSCs. A significantly higher number of anopheline mosquitoes were collected outdoors (69.2%, *n* = 533/770) than indoors (30.8%, *n* = 237/770). There was a statistically significant difference in the mean count of *Anopheles* mosquitoes between outdoors and indoors (t-test, *t*_(931)_ = 8.38, *P* = 0.0001). In the present study, mosquitoes captured in traps in index case households accounted for 13.0% of those captured; in neighboring households, for 54.0%; and in control households, for 33.0%. A higher count of *Anopheles* mosquitoes was observed in index households by both CDC-LTs and PSCs (1.17 mosquitoes per household). A statistically significant difference between groups was determined by ANOVA (*F*_(2930)_ = 4.7,* P* = 0.009) (Table 1). The pair-wise comparison revealed that the *Anopheles* mosquito count was significantly higher in the index case compared to control households (t-test, *t*_(930)_ = 2.5, *P* = 0.01), and neighboring-to-control households (t-test, *t*_(930)_ = 2.71, *P* = 0.006). However, no statistically significant difference was observed between the index and neighboring households (t-test, *t*_(930)_ = 0.66, *P* = 0.050) (Additional file 1: Figure S1).

**Table 1:** *Anopheles* mosquitoes caught in reactive case detection households indoors and outdoors by CDC light traps and pyrethrum spray collection in Arjo Didessa, southwestern Ethiopia, November 2019–October 2021

Household type	PSC indoor, *n* (%)	CDC-LTs indoor, *n* (%)	CDC LT outdoor, *n* (%)	Total *n* (%)	Mean per HH (± SEM)
Index	15 (1.9)	24 (3.2)	102 (13.2)	141 (18.3)	1.17 ± (0.33)
Neighboring	17 (2.2)	132 (17.2)	299 (38.8)	448 (58.1)	0.9 ± (0.14)
Control	13 (1.7)	36 (4.6)	132 (17.2)	181 (23.6)	0.58 ± (0.13)
Grand total	45 (5.8)	192 (25.0)	533 (69.2)	770	0.82 ± (0.10)

Numbers in parentheses are the percentage calculated from the total number of anopheline mosquitoes collected by both methods

*CDC-LTs* U.S. Centers for Disease and Prevention light traps, *HHs* household, *PSC* pyrethrum spray catch, *SEM* standard error of the mean

The morphological identification revealed that the *An. gambiae* complex was the predominant species in the catches, accounting for 87.0% (*n* = 671/770) of the collected mosquitoes, followed by the *An. coustani* complex (12.7%, *n* = 97/770) and*Anopheles* *pharoensis.* 0.26% (*n* = 2/770). Among the collected female anopheline mosquitoes, 72.0% (*n* = 552/770) were unfed, 26.9% (*n* = 226/770) blood-fed and 1.3% (*n* = 10/770) were gravid. Of the total *An. gambiae* s.l., 67.7% (*n* = 454/671) were collected outdoors and 32.3% (*n* = 217/671) were collected indoors; for *An. coustani*, 79% (*n* = 77/97) were collected outdoors and 21% (*n* = 20/97) indoors.

**Fig. 2:**
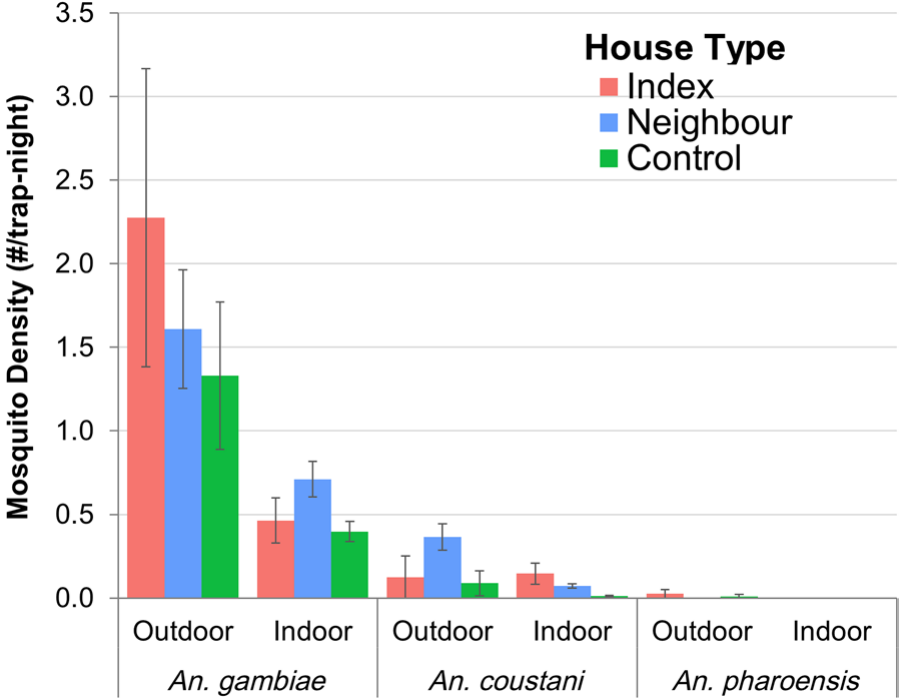
Mean density of collected female anopheline mosquito species indoors and outdoors of RCD households by species using CDC-LTs in Arjo Didessa, Ethiopia. CDC-LTs, U.S. Centers for Disease and Prevention light traps; RCD, reactive case detection

In CDC-LT collections, the overall mean density of anophelines was 1.26 (95% confidence interval [CI] 0.9–1.57) mosquitoes per trap per night. Outdoor traps caught 1.9 mosquitoes per trap night, as compared to 0.64 mosquitoes per trap night for indoor traps. The density of mosquitoes outdoors and indoors was statistically significantly different (t-test, *t*_(574)_ = 4.3, *P* = 0.0001). Comparing the density of mosquitoes in index and neighboring households to control households, the density of anophelines/trap/night in index households was 1.5 (95% CI 0.7–2.3) and in neighboring households was 1.3 (95% CI 0.9–1.7), which were significantly higher (*P* = 0.04) than the density of anophelines/trap/night in control households, which was 0.98 (95% CI 0.4–1.5). The density of *An*. *gambiae* s.l. outdoors was statistically significantly higher than that indoors (t-test, *t*_(574)_ = 3.52, *P* = 0.0004). Similarly, *An. coustani* had a higher density outdoors than indoors (t-test, *t*_(574)_ = 4.3, *P* = 0.0001]. However, there was no statistically significant difference in either species by household type (Table 2; Fig. 2).

**Table 2:** The abundance of *Anopheles* mosquitoes caught by CDC light traps, by type of reactive case detection household in Arjo Didessa, southwestern Ethiopia, November 2019–October 2021

Anopheline species	Household type			Total mean (95% CI)
	Index HH, mean (95% CI)	Neighboring HH, mean (95% CI)	Control HH, mean (95% CI)	
*Anopheles arabiensis*	1.4 (0.4–2.3)	1.1 (0.7–1.6)	0.8 (0.4–1.3)	1.09 (0.8–1.4)
*An. coustani*	0.2 (0.02–0.37)	0.18 (0.1–0.2)	0.18 (0.003–0.2)	0.17(0.1–0.2)
*An. pharonesis*	0.01 (− 0.005 to 0.02)	0.00	0.005 (− 0.005 to 0.01)	0.003(− 0.001 to 0.008)
Overall	1.5 (0.7–2.3)	1.3 (0.9–1.7)	0.98 (0.4–1.5)	1.26 (0.94–1.57)

*CDC-LTs* U.S. Centers for Disease and Prevention light traps, *CI* confidence interval, *HH* household

In PSC collections, the mean mosquito density per house per trap was significantly higher (*P* = 0.001) in index households (0.38 per house per trap, 95% CI 0.201–0.56) than in neighboring households (0.095 per house per trap, 95% CI 0.003–0.180) and control households (0.094 per house per day, 95% CI 0.003–0.191) (*P* = 0.002). There was no statistically significant difference between neighboring and control households (*P* = 0.9) for all species.

### Molecular identification of *An. gambiae* complex

Among the sub-samples of randomly selected *An. gambiae* complex mosquitoes that were morphologically identified (30.0%, *n* = 191/671), 83.8% (*n* = 160/191) belonged to *An. arabiensis*, 7.8% (*n* = 15/191) to *An. amharicus* and the remaining 8.4% (*n* = 16/191) were unamplified (Additional file 2: Figure S2).

### Molecular identification of mosquito host blood meal sources

Blood meal analysis using a cytochrome* b*-based PCR assay was performed on 168 engorged female anophelines, 144 (20.0%) from the CDC-LT collections and 24 (57.0%) from the PSC collections. The DNA of four potential vertebrate hosts (humans, bovines, goats and dogs) was amplified and tested for all blood-fed mosquitoes.

In this study, 30 (17.8%) blood meal samples had a human blood origin, 41 (24.4%) had a bovine blood origin, 14 (8.4%) had a goat blood origin and six (3.6%) had mixed blood meal origins (human, bovine and goat). Only one (0.7%) mosquito was detected whose blood meal origin was dog. Two mosquitoes (1.2%) were found to feed on different types of animals (bovine and goat). *Anopheles arabiensis* showed the widest host range and was found to feed on humans, bovines, goats and dogs. The blood meal indices for *An. arabiensis* was 19.0% (*n* = 28) for human blood, 23.1% (*n* = 34) for bovines, 8.1% (*n* = 12) for goats and 0.7% (*n* = 1) for dogs. Of the total *An. amharicus* identified, 14.2% (*n* = 2) had fed on human blood, 21.4% (*n* = 3) had fed on bovine blood and 7.1% (*n* = 1) had blood-fed on goats. Both *An. coustani* and *An. pharoensis* exclusively feed on animals*.* Interestingly, *Anopheles* species that only fed on human blood were not detected. None of the species identified feed exclusively on human blood. The source of the blood meal in about 44% of the fed mosquitoes could not be identified, suggesting the presence of other sources of blood meal in the study households. The HBI and BBI of *An. arabiensis* were 20.8% and 24.4%, respectively, and those of *An. amharicus* were 1.2% and 1.7%, respectively (Table 3).

**Table 3:** Host blood meal sources of anopheline species with type of reactive case detection households in Arjo Didessa, Southwestern Ethiopia, November 2019–October 2021

Anopheline species	HH type	Total tested, *n*	Single host				Mixed feeding		Other sources, *n* (%)
			Human, *n* (%)	Bovine, *n* (%)	Goat, *n* (%)	Dog, *n* (%)	H + B + G, *n* (%)	B + G, *n* (%)	
*Anopheles arabiensis*	Total	147	28 (19.0)	34 (23.1)	12 (8.1)	1 (0.7)	6 (4.8)	1 (0.7)	64 (43.5)
	Index	40	7	8	3	0	2	0	20
	Neighboring	70	14	21	6	0	3	1	25
	Control	37	7	5	3	1	1	0	19
*An. amharicus*	Total	14	2 (14.2)	3 (21.4)	1 (7.1)	0 (0.0)	0 (0.0)	0 (0.0)	8 (57.0)
	Index	2	1	1	0	0	0	0	0
	Neighboring	6	1	1	0	0	0	0	4
	Control	6	0	1	1	0	0	0	4
*An. coustani*	Total	5	0	2	1	0	0	1	1
	Neighboring	5	0	2	1	0	0	1	1
*An. pharoensis*	Total	2	0	2	0	0	0	0	0
	Neighboring	2	0	2	0	0	0	0	0
Grand total		168	30 (17.8)	41 (24.4)	14 (8.4)	1 (0.6)	6 (3.6%)	2 (1.2%)	74 (44.0)

Values in the table are given as the number (*n*) of blood meal sources, with the percentage in parentheses

*H + B + G* Human + bovine + goat, *B + G* bovine + goat, *HH* household

**Fig. 3:**
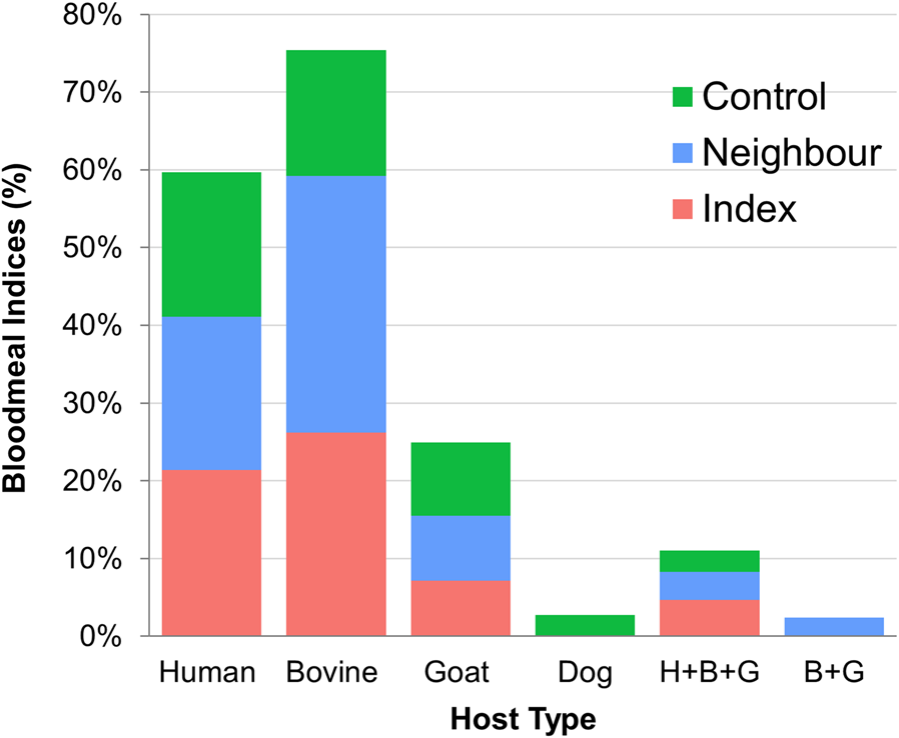
Host blood meal sources of *Anopheles* mosquitoes in index, neighboring and control households. H + B + G, Human + bovine + goat blood meal source; B + G, bovine + goat blood meal source

Most notably, there was no significant difference in number of human blood meals between all anopheline mosquitoes and *An. arabiensis* by household type (Chi-square test, *χ*^*2*^ = 0.3, *df* = 2*, P* = 0.8 and *χ*^*2*^ = 0.1, *df* = 2*, P* = 0.9, respectively). On the other hand, mosquitoes that fed on animal blood did show a statistically significant difference (Chi-square test, *χ*^*2*^ = 5.6, *df* = 2*, P* = 0.05) between household types, namely 26.2%, 36.1% and 16.2% in the index, neighboring and control households, respectively. The BBI of *An. arabiensis* was statistically significantly different between the index, neighboring and control households (*χ*^*2*^ = 6.1, *df* = 2*, P* = 0.04) (Table 3; Fig. 3).

Among the blood-fed *An. arabiensis*, the majority (68.0%, *n* = 100/147) were caught indoors, and 32.0% (*n* = 47/147), were caught outdoors. Human DNA was detected in 55.8% (*n* = 19/34) of *An. arabiensis* that was collected indoors and in 44.2% (*n* = 15/34) of those caught outdoors. All human blood-fed *An. amharicus* were caught indoors. Out of 54 animal blood-fed mosquitoes, 38 (70.4%) and 16 (29.6%) were *An. arabiensis* which were caught from indoors and outdoors, respectively. The remaining 10 (18.5%) animal blood-fed mosquitoes were *An. coustani*, *An. amharicus* and *An. pharoensis*, which were caught from outdoors.

### Mosquito *Plasmodium* sporozoite infections

Among the 770 female anophelines tested with CSP-ELISA, 4.4% (*n* = 34/770) were positive for *Plasmodium* CSPs. From the total positive sporozoites, 2.3% (*n* = 18/770) were *P. vivax* and 2.1% (*n* = 16/770) were *Plasmodium falciparum* infections. More than half (53.0%) (*n* = 18/34) of those were positive for *Pv-247 and Pv-210*, while 47.0% (*n* = 16/34) were positive for *Pf* sporozoites*.* All sporozoite-positive mosquitoes were molecularly identified to the species level, and were found to be *An. arabiensis*. The overall sporozoite (*Pf, Pv-210 and Pv-247*) rate for *An. arabiensis* was 4.8% (*n* = 32/671). Of the CSP-positive mosquitoes, 94.1% (*n* = 32/34) were *An. arabiensis*, while 2.1% (*n* = 2/97) were *An. coustani,* which was positive for *Pv-247*. Interestingly, none of the *An. amharicus* and *An. pharoensis* samples were found to be positive for CSPs (Table 4).

**Table 4:** The sporozoite infection rate of *Anopheles* mosquitoes in reactive case detection households, Arjo Didessa, southwestern Ethiopia, November 2019–October 2021

Household type	*Anopheles arabiensis*				*Anopheles coustani*				Overall, *n* (%)
	Total tested,* n*	*Pf,* *n* (%)	*Pv*-247, *n* (%)	*Pv*-210, *n* (%)	Total tested,* n*	*Pf*, *n* (%)	*Pv-*247, *n* (%)	*Pv*-210, *n* (%)	
Index HHs	124	5 (4.03)	2 (1.61)	1 (0.08)	16	0 (0.00)	0 (0.00)	0 (0.00)	8 (5.71)
Neighboring HHs	388	11 (2.83)	11 (2.83)	2 (0.51)	60	0 (0.00)	0 (0.00)	0 (0.00)	24 (5.35)
Control HHs	159	0 (0.00)	0 (0.00)	0 (0.00)	21	0 (0.00)	2 (9.52)	0 (0.00)	2 (1.11)
Total (*n* = 770)	671	16 (2.38)	13 (1.93)	3 (0.44)	97	0 (0.00)	2 (2.06)	0 (0.00)	34 (4.41)

*CSP* Circumsporozoite protein, *HHs* households, *Pf Plasmodium falciparum*, *Pv-247* *Plasmodium vivax*-247 CSP, *Pv-210* *P. vivax*-210

Of these CSP-positive mosquito samples, 58.9% (*n* = 20/34) were captured indoors, and 41.1% (*n* = 14/34) were captured outdoors. The sporozoite rate was higher indoors (8.4%, *n* = 20/237) than outdoors (2.6%, *n* = 14/533), with a statistically significant difference (Fisher’s exact test, *χ*^2^ = 13.2, *df* = 1, *P* = 0.0002) (Additional file 3: Figure S3). In addition, the sporozoite rate varied according to the site (irrigated vs non-irrigated) and season (dry vs wet). However, no statistically significant difference was observed regarding these variables.

**Fig. 4:**
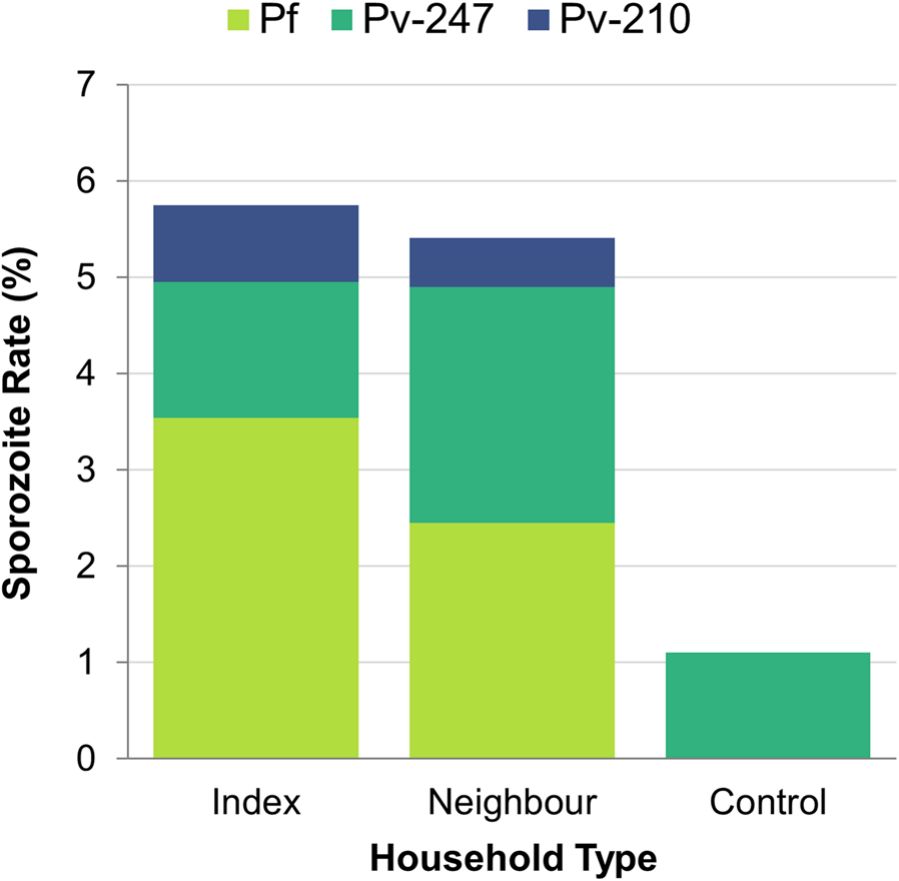
*Anopheles* mosquito sporozoite infection rates in reactive case detection study households, Arjo Didessa, Southwestern, Ethiopia. *Pf, Plasmodium falciparum* circumsporozoite protein (CSP); *Pv*-247, *Plasmodium vivax*-247 CSP; *Pv*-210, *P. vivax*-210

The proportion of CSP-positive mosquitoes in index, neighboring and control households was 5.7% (*n* = 8/141), 5.3% (*n* = 24/448) and 1.1% (*n* = 2/181), respectively. There was a significant difference in the proportion of CSP-positive mosquitoes collected from index households compared to control households (Chi-square test, *χ*^*2*^ = 5.5, *df* = 1*, P* = 0.02) and from neighboring household compared to control households (Chi-square test, *χ*^*2*^ = 5.6, *df* = 1, *P* = 0.01). However, there were no significant differences in the proportion of CSP mosquitoes collected from the index and neighboring households (Chi-square test, *χ*^*2*^ = 0.02, *df* = 1, *P* = 0.08) (Fig. 4).

## Discussion

The results of the present study showed a higher outdoor mosquito density and higher sporozoite-infected anopheline vector rate in malaria index cases and neighboring households, compared to control households, which could contribute to the ongoing malaria transmission in the study area. In this study, *An. arabiensis* was the most predominant species, followed by *An. coustani*. *Anopheles arabiensis* had the higher *Plasmodium* infection rate. Also, *An. arabiensis* showed a wider host range and was found to feed on humans, bovines, goats and dogs. Remarkably, the study found that *An*. *amharicus* fed on human blood and *An. coustani* was infected with sporozoites.

The higher outdoor density of mosquitoes in the present study may be attributed to long-term indoor-based vector control interventions. In addition, this increasing outdoor density of mosquitoes might be associated with water resource developments [37–39], overwatering of seedlings [39], outdoor activities and population movements [40]. Also, it could be a change in vector behavior in response to IRS spraying in districts of the region [29]. In comparison, in control households of this study, no statistically significant variation in the density of mosquitoes was observed from that in the index and neighboring households. This is more likely to be a result of the distance between households in the mosquito’s flight range to search for the host [41, 42].

*Anopheles arabiensis*, the primary malaria vector in the area, showed higher outdoor density around malaria index cases and neighboring households, which could increase outdoor blood-feedings at dawn and dusk while people are outside working at agricultural tasks [43]. In addition, this may be due to the prolonged application of insecticidal vector control measures, which cause behavioral changes in mosquitoes [17, 44]. The higher number of outdoor mosquitoes may also substantially increase and maintain residual malaria transmission [45]. Thus, this finding may suggest the necessity of other supplementary vector control interventions targeting outdoor mosquitoes, which have changed their biting and resting behavior from indoors to outdoors in response to indoor interventions [46, 47].

In the present study, animal DNA was detected proportionally higher than human DNA in blood-fed *Anopheles* mosquitoes (34.5% vs 17.8%, respectively). *Anopheles* mosquitoes have a wide range of blood meal sources in animals, as indicated by their lower HBI compared to their BBI. Consistent with the results of the present study, *An. arabiensis* has been shown in previous studies to be opportunistic in terms of blood meal sources [48, 49]. This diversified zoophilic and anthropophilic behavior could also be associated with indoor-induced vector control interventions [44]. These diverse mosquito behaviors may also be contributing to maintaining residual transmission [45]. However, extrinsic factors, such as the availability of a host, as well as intrinsic factors, such as sibling species of mosquitoes, may influence a vector’s selection of a host [50]. In general, this finding of a higher blood-feeding rate on animals than on humans could represent an opportunity to tackle residual malaria transmission by introducing animal-based interventions [51, 52].

On the other hand, while overall there was a reduced tendency for feeding on human blood, most blood-fed mosquitoes were found indoors. A high proportion of blood-fed *An. arabiensis,* 68%, was detected indoors in this study. This high proportion of indoor blood-fed mosquitoes could be the consequence of the emergence of insecticide resistance by *An. arabiensis* [11]. Our findings are supported by those of a previous study in which pyrethroid resistance was found in *An. arabiensis* in the study area [14]. Thus, it is important to strengthen vector control interventions such as targeted IRS and LLINs, as well as continue monitoring the insecticide resistance status in the study area.

Sporozoite-positive *An. arabiensis* were detected in the study households both indoors and outdoors. Significantly higher infection rates were found indoors than outdoors, suggesting ongoing malaria transmission in the study area. In line with the results of other studies [53, 54], *An. arabiensis* was found to be sporozoite-positive outdoors. This sporozoite infection rate outdoors might be attributed to changes in the biting behavior of *An. arabiensis* [9]*.* Outdoor mosquitoes accounted for 70% of the *Anopheles* mosquito population in this study. As result, the sporozoite-positive outdoor mosquitoes, together with a higher density of outdoor mosquitoes and increased insecticide resistance in the study area, could sustain residual malaria transmission. The higher infection rates outdoors may represent a major threat to vector control tools that only target indoor malaria transmission. Thus, supplementary vector control interventions together with the existing vector control measures are needed to control outdoor malaria transmission [55].

In comparison to previous studies conducted in Ethiopia, the overall sporozoite rates for *P. falciparum* and *P. vivax* in the present study were higher [56–58]. Importantly, more sporozoite-carrying mosquitoes were captured from the households of the index cases and neighbors. In line with this finding, sporozoite infection rates have been previously shown in index and neighboring households in low transmission settings [54]. It should be noted that there were 5.6%, 5.2%, and 1.2% sporozoite infections in the index, neighboring and control households, respectively, in the present study. Control households had the lowest sporozoite infection rates, possibly due to their distance from malaria index case households. Additionally, our study demonstrated that *An*. *arabiensis* had a relatively higher sporozoite infection rate and may be contributing to ongoing malaria transmission in the index cases and their immediate neighboring households.

In the present study, we identified a higher sporozoite rate in index cases and their immediate neighboring households compared to those reported in other malaria-endemic region studies in Ethiopia [59–61]. This result may provide evidence of where to target interventions to interrupt residual malaria transmission. Moreover, our findings provide a plausible reason for why core vector control interventions should target small geographical areas, such as houses, villages and hotspots, rather than the wider community to achieve malaria elimination.

It was remarkable that *An*. *amharicus* demonstrated the plasticity of feeding on both humans and animals in our investigation. In addition, this species has been reported to show resistance to pyrethroids in the study area [14]. As a result, the propensity of *An. amharicus* to feed on human blood could eventually enable it to be a potential vector of malaria [18]. However, despite this tendency of *An. amharicus* to feed on human blood, all specimens tested negative for *Plasmodium* sporozoites in this study. *Anopheles* *amharicus* accounted for approximately 8.0% of the total population of the *An*. *gambiae* complex. Given its propensity to feed on human blood, further study is needed to determine its role in malaria transmission in the study area.

The current study showed that *An*. *coustani* and *An. pharoensis* had an exclusive zoophilic tendency, as reported earlier by the authors of previous studies conducted in southwest Ethiopia [53, 57]. Studies have implicated *An. coustani* as a vector for the transmission of malaria, which is consistent with our findings [56, 62] and in line with the results of a study from Zambia in which sporozoite positivity in *An. coustani* was detected in low transmission settings [54]. In addition, *An. coustani* reared in wild under laboratory conditions demonstrated a susceptibility to *P. falciparum* and *P. vivax* infections [63]. In our study, *An. coustani* was found to have higher outdoor densities and sporozoite positivity. In this context, *An. coustani* plays a large role in outdoor malaria transmission [64], and the interruption of residual malaria in this context may also be more challenging.

In general, to achieve success with elimination efforts, studies also have suggested a combination of vector control interventions, such as intensifying LLINs, IRS, modified outdoor trapping, animal-based interventions, improved housing and local larva control, for both indoor and outdoor malaria transmission control [17, 55, 65].

In the present study, the presence of a higher outdoor density of *An. arabiensis,* coupled with sporozoite-infected mosquitoes outdoors, could be a major threat to achieving success in malaria elimination. Therefore, routine vector control might not be effective in targeting outdoor malaria transmission. In addition, the presence of indoor malaria vectors with sporozoite positivity, which is also responsible for indoor malaria transmission, was also detected in this study. This result implies that in the study area there could be an emergence of insecticidal resistant malaria vectors, a failure in the implementation of core indoor vector control interventions and ongoing malaria transmission.

In conclusion, the results of this study largely suggest that vector control interventions, such as targeted IRS, LLINs and other supplementary vector control interventions (e.g. larval source management and community engagement) are needed to targeting malaria index cases and their neighboring households. Furthermore, implementing environmental management for targeted vector control, particularly larval control, will reduce mosquito density. Also, case-based surveillance to identify transmission foci, vector surveillance in search of persistent vector populations and monitoring of insecticide resistance status are needed to interrupt residual malaria transmission and achieve malaria elimination efforts.

This study's main limitations are that the most advanced detection technique (PCR) was not used to determine sporozoite rates, and that the characteristics, habitat and density of larvae were not investigated.

## Conclusions

The findings of this study indicated that malaria index cases and their neighboring households had higher outdoor mosquito densities and *Plasmodium* infection rates. The study also highlighted a higher outdoor mosquito density, which could increase the potential risk of outdoor malaria transmission and may play a role in residual malaria transmission. Thus, it is important to strengthen the implementation of vector control interventions, such as targeted IRS, LLINs and other supplementary vector control, including larval source management and community engagement approaches. Furthermore, in low transmission settings such as the Arjo Didessa Sugarcane Plantation, providing health education to local communities, enhancing environmental management and implementing entomological surveillance, along with case detection and management by targeting malaria index cases and their immediate neighboring households, could be important measures by which to control residual malaria transmission and achieve the targeted elimination goals.

### Supplementary Information

**Additional file 1: Figure S1. **The mean density of* Anopheles *mosquitoes in the index, neighboring, and control HHs in Arjo, Didessa, Ethiopia.

**Additional file 2: Figure S2.** Molecular identification of *An. gambiae* species complex collected from RCD study households in Arjo Didessa, Ethiopia.

**Additional file 3: Figure S3.** Sporozoite infection rate in indoor and outdoor of RCD study households, Arjo Didessa, Southwestern Ethiopia.

## Data Availability

The datasets used in this study are available upon reasonable request from the corresponding author. The data supporting the study are available within the article and/or its supplementary materials, or deposited in a publicly available database.

## Abbreviations

CSP: Circumsporozoite protein
ELISA: Enzyme-linked immunosorbent assay
ICEMR: International Center Excellence for Malaria Research
RCD: Reactive case detection
TIDRC: Tropical Infectious Diseases Research Center
