# Supplementary figures and images for: Higher outdoor mosquito density and Plasmodium infection rates in and around malaria index case households in low transmission settings of Ethiopia: Implications for vector control
Source: Parasit Vectors. 2024 Feb 6;17:53. doi: 10.1186/s13071-023-06088-2 (PMC10848356; doi:10.1186/s13071-023-06088-2)

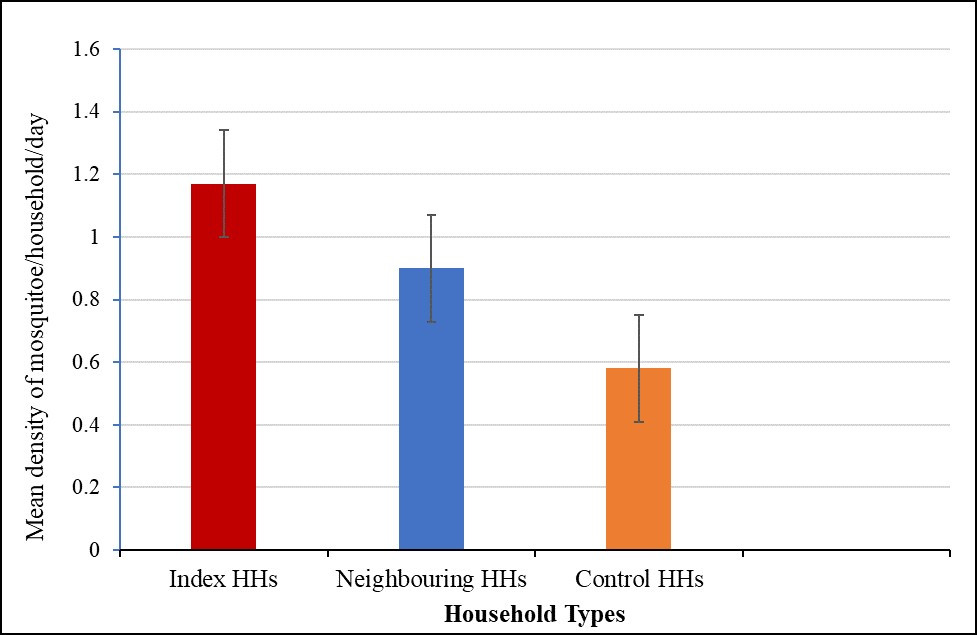

Supplement: Supplementary file 1 — Additional file 1: Figure S1. The mean density of Anopheles mosquitoes in the index, neighboring, and control HHs in Arjo, Didessa, Ethiopia. [file 13071_2023_6088_MOESM1_ESM.tif]

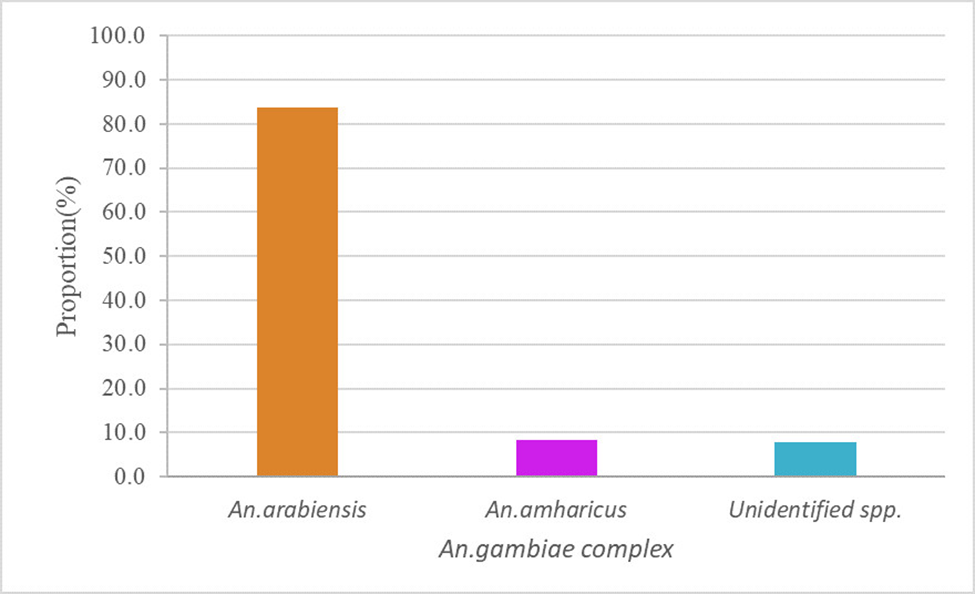

Supplement: Supplementary file 2 — Additional file 2: Figure S2. Molecular identification of An. gambiae species complex collected from RCD study households in Arjo Didessa, Ethiopia. [file 13071_2023_6088_MOESM2_ESM.tif]
